# Hospital Resilience After the 2015 Earthquake in Nepal: Results From Semi-structured Interviews With Hospital Staff

**DOI:** 10.3389/fpubh.2021.602509

**Published:** 2021-02-26

**Authors:** Maria Moitinho de Almeida, Joris Adriaan Frank van Loenhout, Sunil Singh Thapa, K. C. Kumar, Deepak Prakash Mahara, Debarati Guha-Sapir, Isabelle Aujoulat

**Affiliations:** ^1^Centre for Research on the Epidemiology of Disasters, Institute of Health and Society, Université Catholique de Louvain (UCLouvain), Brussels, Belgium; ^2^Department of Orthopedics, Tribhuvan University Teaching Hospital, Kathmandu, Nepal; ^3^Tribhuvan University Teaching Hospital, Kathmandu, Nepal; ^4^Institute of Health and Society, Université Catholique de Louvain (UCLouvain), Brussels, Belgium

**Keywords:** disaster, health service research, earthquake, hospital resilience, qualitative research, hospital staff

## Abstract

**Background:** Resilient hospitals are increasingly recognized as a cornerstone of disaster reduction in global policies such as the Sendai Framework for Action. However, current hospital resilience frameworks emerged from pre-disaster conceptualizations, and have not been verified in real-life disaster contexts nor in the frontlines. Our aim was to study a tertiary hospital's resilience after the 2015 earthquake in Nepal, as experienced by its staff.

**Methods:** We undertook a qualitative study in the Tribhuvan University Teaching Hospital (TUTH), where we conducted 18 semi-structured interviews with hospital staff. We inductively created themes to describe the earthquake burden to the hospital, and to analyze individual resilience of hospital staff. In addition, we deductively documented the resilience of the hospital as a system, according to the system resilience dimensions: means of resilience (redundancy and resourcefulness), and ends of resilience (robustness and rapidity).

**Results:** In terms of robustness, TUTH increased its capacity for earthquake victims as elective activities were temporarily interrupted and quality of care was not a priority. Three stages of rapidity were identified: critical rapidity to address immediate needs, stabilizing rapidity until the hospital re-started routine activities, and recovery rapidity. In addition to the disaster plan, emerging adaptations played a major role in redundancy and resourcefulness. We found that individual resilience depended on three determinants: safety, meaningfulness, and sense of belonging.

**Conclusions:** Hospital resilience results from a complexity of emerging and planned adaptations, as well as from interdependencies with individual resilience. Frameworks and plans to improve hospital resilience must reflect flexibility of response, and a concern for well-being of hospital staff is central for sustainable disaster response and improved resilience.

## Introduction

Disasters are events that lead to substantial losses and disrupt the functioning of a community beyond its coping capacity (1). Large scale, sudden-onset disasters occur quickly or unexpectedly, cause widespread destruction, produce high numbers of deaths and injuries, and require external assistance (1). Countries with increased vulnerability and weaker coping capacity are the most affected by the human consequences of such large-scale disasters, which heavily strain health systems by causing sudden material damages, increases in demand, or workforce disruptions (2–6). The role hospitals play in an effective community disaster response has been increasingly recognized (7, 8), and resilient hospitals have become central in global disaster risk reduction initiatives. For instance, the Hyogo Framework for Action 2005–2015 (9) described the processes needed to reduce disaster risks in various sectors, including the health sector, and the more recent Sendai Framework for Action 2015–2030 (10) provides recommendations for safe cities and hospitals, and calls for resilient national health systems and critical infrastructures.

Despite a growing recognition of its importance, the concept of hospital disaster resilience remains elusive (11). Initially developed by engineering sciences, the concept of hospital resilience describes the “ability to overcome disasters with inherent capacity and adaptive flexibility, providing emergency medical services and responding to sudden increases in demand, while retaining essential functionalities” (7). The four “resilience R's” framework of hospital resilience consists of “means” of resilience—Redundancy and Resourcefulness—and “ends” of resilience—Robustness and Rapidity. Redundancy is the extent to which elements are substitutable, including the hospital itself if linkages are in place. Resourcefulness is the ability to mobilize material and human resources to meet priorities and achieve goals, including coordination measures. Robustness is the strength to withstand stress without suffering loss of function. Rapidity entails that priorities are met in a timely manner, in order to contain losses, recover functionality, and avoid further disruption (12).

Building on existing frameworks, instruments that assess hospital resilience have been recently developed, but they only consider pre-disaster states (13, 14); it remains unclear whether they reflect processes in the context of actual disasters. A recent paper attempted to use existing frameworks after a disaster, but it focused on experiences at managerial levels rather than from the frontlines (11). However, frontline staff are confronted with difficult situations that are often not reflected in strategic level experiences, but greatly influence overall disaster response and, ultimately, disaster impact. Only by understanding experiences of hospital staff can we design optimal plans that foster hospital resilience. In this paper, we study a tertiary hospital's resilience after the 2015 earthquake in Nepal, as perceived by its frontline staff.

## Methods

### Study Setting

On April 25th 2015, a powerful earthquake hit Nepal, killing nearly 9,000 people. It was followed by recurrent aftershocks, the strongest on May 12th (15, 16).

Our study focuses on a referral tertiary hospital in Kathmandu: the Tribhuvan University Teaching Hospital (TUTH). TUTH was established with donation funds from the Japanese government, has earthquake-resistant infrastructure, and is centrally located and easily accessible. Initially a 300 bed-capacity, it expanded over the years reaching 700 beds in 2018, but nearly a hundred additional beds were created after the earthquake in 2015 (17). As a University hospital, TUTH contributes to training and education of medical and healthcare professions, in addition to providing tertiary and specialized care to the population of Nepal. TUTH was part of the Hospital Preparedness for Emergencies (HOPE) initiative, a training course that addresses the structural, non-structural, organizational, and medical concerns of health facilities in order to design and implement effective disaster response plans (18). Consequently, at the time of the 2015 earthquake, TUTH had an emergency plan in place. Hospital staff were trained in the implementation of this plan through regular drills and other physical measures had been implemented, such as supply and maintenance of emergency containers, electricity replacement, and fixing of objects (18). TUTH was one of the seven hub hospitals in the Kathmandu valley treating earthquake victims and coordinated with other health facilities for referral. TUTH's direction immediately activated the Incident Command System, and a triage system for mass casualty incidents was put in place. TUTH treated 1,723 victims in total (19), and more than 500 were admitted, mostly with orthopedic injuries and surgical needs (20). Non-injury-related admissions were significantly lower in the weeks after the earthquake, as priority was given to resource-intense earthquake injuries (21).

### Data Collection

The first author, a non-Nepalese female Medical Doctor, conducted 18 semi-structured interviews with hospital staff to explore their experiences of hospital resilience after the earthquake. Interviewees were purposively selected to obtain a diverse sample in terms of profession, gender, and seniority. The sample size was estimated according to available resources, but data saturation was reached on the 14th interview, meaning that no new information emerged from the later interviews (22). The hospital director and/or administrative chief invited interviewees to participate directly or through their direct supervisors, who gave them permission to participate. Participants had no previous contact with the interviewer.

The interviewer followed an interview guide that focused on experiences before, during, and after the earthquake at personal, professional, and organizational levels (Supplementary File 1). Different probes were used to encourage interviewees to elaborate on certain aspects that merited to be explored more in-depth.

Interviews took place in May 2018 in a quiet room at TUTH during working hours. For seven interviewees less comfortable in expressing themselves in English, an external interpreter facilitated communication. Interviews lasted on average 60 min, ranging between 40 and 100 min.

At the start of each interview, study participants were informed about the researcher's background and the content of the research. They were given the opportunity to read the informed consent form before signing it. Upon participants' approval, all interviews were audio recorded, transcribed ad verbatim, and, if applicable, translated to English. Notes were taken during the interviews, and were incorporated in the initial coding. Interview transcripts were returned to participants, but no changes were requested.

### Data Analysis

Following recommendations for qualitative data analysis in health services research, we combined deductive and inductive thematic coding (23). The first author coded the data, and themes were discussed among the research team.

We first inductively created themes to describe the earthquake burden to the hospital, as perceived by the respondents. Then, in order to document the resilience of the hospital as a system, we extracted parts of the data that thematically aligned with the four dimensions of hospital resilience, the theoretical framework that defined our starting codes: Redundancy, Resourcefulness, Robustness, and Rapidity. Some hospital adaptations could be categorized in multiple dimensions. We followed the definitions proposed in the literature (7, 12), but establishing clear distinctions was sometimes difficult. In such cases, we categorized the content in multiple dimensions and explain how it aligns with each.

Finally, we found that individual resilience of hospital workers was an important component of hospital resilience, but could not fit in the “4R's” since they are conceived for systems, not individuals. We undertook inductive coding to analyze individual resilience of hospital staff, and three themes emerged: safety, meaningfulness, and sense of belonging.

We handled and coded the data using NVivo software, 12th edition. In our results, we present quotations to support our statements, some of which were slightly modified for clarity. Because interviews were conducted in one hospital and touched sensitive issues, we present quotations without participant information to prevent identification. To ensure maximum transparency and reproducibility of the findings, this study complies with the Consolidated Criteria for Reporting Qualitative Research (COREQ) (24).

## Results

### Characteristics of Study Participants

We interviewed 18 hospital staff of different functions and demographic characteristics, presented in Table 1.

**Table 1:** Characteristics of interviewees.

**Characteristics**	**Number**
**Profession**	
Medical Doctor (Departments: orthopedics, gastroenterology, anaesthesiology, emergency department)	4
Nurse (Departments: intensive care unit, operation theater, maternity ward, administration)	4
Physiotherapist	2
Pharmacist and dietician	2
Radiology and laboratory technician	2
Security and housekeeping	2
Finance and administration	2
**Gender**	
Male	11
Female	7
**Age Group (at time of the interview)**	
30–40	7
41–50	4
51–60	7

Based on the information collected in the interviews, we identified four types of burden to the hospital: material challenges, challenges to health service provision, challenges to management and coordination, and emotional and physical impact on individuals. Table 2 expands on these burdens, and presents adaptations at system level under “means of resilience,” whereas the resulting robustness and rapidity are the “ends” of resilience. Table 2 also presents the main determinants of individual resilience.

**Table 2:** Overview of earthquake burdens and system and individual resilience based on information from the interviews.

**Burden**	**System resilience (the “4 R's”)**			
	**“Means” of resilience**		**“Ends” of resilience**	
	**Redundancy**	**Resourcefulness**	**Robustness**	**Rapidity**
***Material damages*** • **Structure** • **Electricity** • **Equipment and supplies**	• Offices in damaged area moved to resistant part • Electricity generator, fuel for 24 h	• Pre-existing disaster plan: use of open-space • Army intervened for fuel supply • Rationing and reutilization of equipment • External donations BUT challenges with storage and validity	• Structure resisted despite minor damages • Objects fell as fixing and retrofitting not widespread • Emergency activities continued	• Critical rapidity: Ability to function immediately • Challenges to critical and stabilizing rapidity: Management of equipment may have caused delays
***Health service provision*** • **Massive influx of earthquake victims** • **Chronic disease patients** • **Mental health (MH)**	• Linkages with other health services	• Emergency triage • Free healthcare for victims • Interruption of elective activities, early discharges • Rearrangement of surgical and ICU services • Long working hours	• Increased capacity for EQ victims • Decreased quality of care? • Health needs addressed	• Critical Rapidity: Surgeries within 2 h • Stabilizing Rapidity: 2–3 weeks to address all victims
	• Task shifting			
***Management and coordination*** • **Saturday and mostly junior** • **“Chaos”** • **Inflow of external organizations, with infiltration of thieves and fraudulent schemes in the crowd** • **Tension with foreign medical teams** • **Second earthquake**		• Establishment of assessment meetings and decision channels (although unclear for some) • Unclear coordination • Spontaneous individual decisions by individuals • Transfer of burden to external actors • Direct requests of foreign teams to the government Second earthquake: • Improved coordination • Prepared staff	• Victims were assessed and surgeries occurred • Hospital able to focus on core activities • Decreased quality of care? • Unclear if following disaster plan Second earthquake: • Improved response	• Benefits to critical and stabilizing rapidity: external support • Challenges to critical rapidity: confusing decision-making and coordination Second earthquake: • Quicker response: limited impact on stabilizing rapidity
***Emotional and physical impact to individuals*** • **Concern with family** • **Feelings of “torture”** • **Exposure to suffering** • **Increased workload**		• Split groups so everyone had time to check on family	• Staff showed up and ensured continuous work	• critical rapidity influenced by contact with family and private life adaptations • Recovery rapidity influenced by personal experiences
	**Individual resilience**			
	Safety	Meaningfulness		Sense of belonging
	• Important to keep working despite aftershocks • Absence of national policies and safety measures contribute to persistence of fear	• Making sense of the tireless work and prioritizing it over family • Dependent on job visibility		• Support from families, co-workers and communities • Key to focus on work without concerns

*These findings are based on a mixed inductive and deductive coding of interviews conducted with hospital staff in a tertiary hospital in Nepal that explored their experiences of hospital resilience. The deductive coding followed the 4R System Resilience framework: Redundancy, Resourcefulness, Robustness, and Rapidity. EQ, earthquake*.

**Figure 1:**
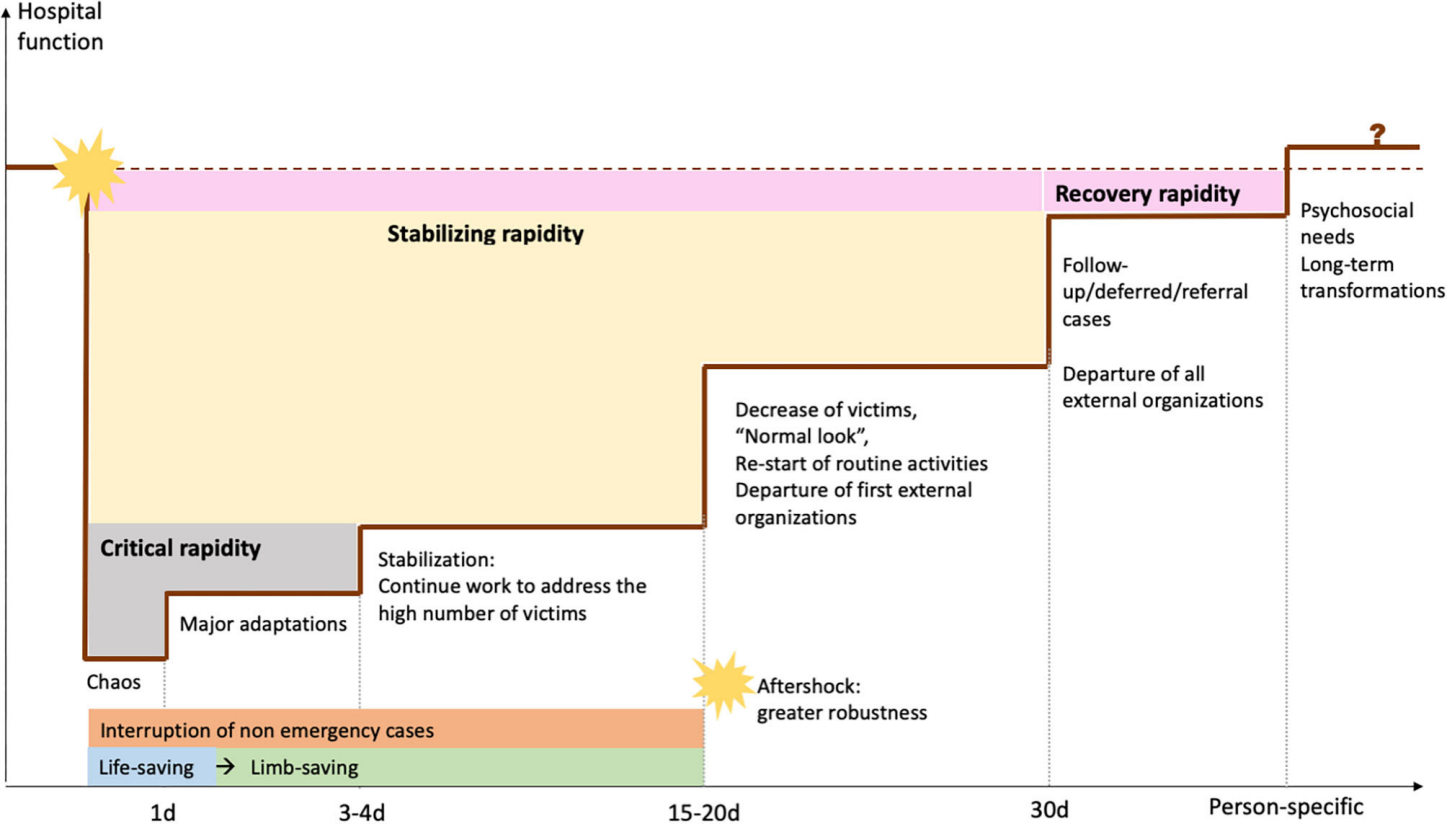
Diagram relating hospital functionality, adaptations, and time, based on information from interviews.

To summarize system resilience at TUTH, Figure 1 illustrates how hospital functionality evolved with adaptations and over time. We briefly summarize the main findings on the ends of resilience (robustness and rapidity) in the following section, followed by a more expanded description of the means of resilience as well as individual resilience.

### Ends of System Resilience

In terms of hospital robustness, TUTH avoided total loss of function and maximized capacity to provide emergency treatment, though at the expense of routine activities and with potential collateral impacts: loss in quality of care and patients discouraged to travel to TUTH even after re-start of routine services. For instance, deliveries at TUTH reportedly decreased with the earthquake and remained low, possibly because women preferred closer health facilities.

*During that time we were not focusing on quality of care. (…) We had a lot of wound infections, we were not taking care of sterility properly… We just needed to provide care, we were focusing on life-saving and limb-saving activities*.

In our analysis of hospital rapidity, we identified three major types of rapidity, as TUTH regained different functions at different times:

- Critical rapidity: the time needed to start essential work and assist injured victims while also self-organizing. At TUTH, the first surgeries started 2 h after the shake, but more days were needed to optimize coordination, since being a Saturday challenged critical rapidity as most senior staff was absent.
- Stabilizing rapidity: the new rhythm after critical reorganizations to address earthquake-related surges, until the hospital re-started routine activities and reobtained a “normal look.” The major aftershock seemed to have had limited impact.
- Recovery rapidity: After the situation was stabilized and the hospital re-started routine activities, time was needed to end the phase—and feeling—of emergency, which was very subjective. Administrative staff and those caring for earthquake victims reported longer times to recovery. Although they often mentioned it, interviewees struggled to elaborate on the concept of “recovery”—some even referred that they were “*still recovering, and not recovered*.”

### Means of System Resilience

#### Redundancy

TUTH found suitable alternatives to disrupted elements. While redundancies to certain challenges were established prior to the earthquake, others emerged as new problems arose.

##### Redundancy to Material Challenges

Services from slightly damaged facilities moved to earthquake-resistant areas, and the fuel-supplied generator provided electricity as central supply was interrupted. However, it only had a 24-h autonomy, and Kathmandu was facing a fuel shortage. To solve this, TUTH coordinated with the army to ensure fuel supply (which demonstrates resourcefulness—see further).

##### Redundancy to Health Service Provision

After the earthquake, “step-down centers” rehabilitated and cared for patients no longer requiring hospital care. TUTH staff actively looked for patients they could transfer in order to accommodate more victims, and a mobile team ensured patient follow-up outside the hospital.

The concept of step down center came immediately. On the second or third day we started sending the patients. (…) We used to go on a vehicle to take a round of all patients. “This patient is serious, we can bring him back to our hospital. This patient is fine, please carry on.”

Reports regarding referrals from TUTH to other hospitals were conflicting: some were unaware they existed, while others affirmed they were in place but patients were reluctant to move to unsafe buildings.

This hospital wanted to refer, and those doctors they wanted to take the patients. But those patients would say “that structure is so scary, we don't want to go to that hospital!”

Staff conducted tasks that differed from their usual work or qualifications. Task shifting could be a feature of both redundancy and resourcefulness; we considered redundancy when hospital workers explicitly switched tasks to replace overwhelmed colleagues.

The surgeons were limited in number, they had to go to the OT (…).So we did the dressings, and we were helping them in keeping the records and discharging the people…

#### Resourcefulness

TUTH was resourceful in mobilizing external and internal resources. Some aspects were specified in the disaster plan, but many adaptations were spontaneous, compensating for a perceived lack of coordination.

##### Resourcefulness to Material Challenges

Despite having containers with emergency supplies, TUTH faced shortages of essential supplies. Interviewees reported rationing and reusing equipment, but also that such strategies could be harmful. Fortunately, there was a massive inflow of external donations. Even if storage was initially challenging, TUTH handled donated surgical and rehabilitation equipment easily; donated medicines were more complicated to manage due to expiration date. Charity groups prepared meals to distribute among staff, patients, and patient families, removing a substantial burden from TUTH.

##### Resourcefulness to Health Service Provision

TUTH immediately started mass casualty triage, consisting of four categories: green (minor injuries), yellow (patients who did not require admission), red (severe injuries), and black (deceased). Only red cases received immediate and/or surgical care.

Elective services were halted, stable non-earthquake patients were discharged earlier than in normal circumstances, operation theaters were continuously functioning, and care for earthquake victims was free of cost. Neurosurgery and orthopedic surgery monopolized operation theaters, and post-operative rooms were rearranged to accommodate more intensive care beds. Staff reorganized duties and worked longer hours to ensure uninterrupted services.

We categorized task shifting as resourcefulness when staff adapted tasks to address new health needs or high patient numbers. For instance, non-medical staff performed first aid, applied plasters and even resuscitated patients; and doctors and nurses conducted psychological counseling during unrelated consultations when they identified mental health needs.

Interviewees were satisfied with volunteer work from patient relatives, local communities, and students. Proximity to the University facilitated the integration of students in hospital work, but this required additional supervision. Nevertheless, foreign surgical teams from western countries were unwelcome, since their presence was felt as undermining TUTH's competence, and interviewees felt they were rather needed in places without health facilities. To handle the inflow of foreign medical teams, TUTH's administration requested that they first registered with the government. Nevertheless, foreign teams who supported in psychosocial counseling were perceived as useful and well-accepted.

We told them “we have our own doctors, we have our own nurses, we are enough already here. You are needed somewhere else. (….) Where the incident took place, there are no medical facilities, you are needed there”. (…) We said “Enough right now. If you want to serve, you have to come through the government.”

*I'm thankful to these volunteers who came from abroad. One young doctor came from Canada and she went to different schools to give psychosocial counseling. Psychosocial skills and counseling were very important*.

##### Resourcefulness to Management and Coordination

Some interviewees believed the day of the earthquake was well-managed, despite reports of a highly chaotic situation. After the first day, the director's team held regular assessment meetings and gave instructions to different departments. The presence of external donors and volunteers also strained TUTH as it led to accountability issues, overflow of supplies, abuse of charity, and theft. Hence, coordination was essential. Most interviewees underlined the importance of having, or finding, a role after the earthquake, but not all were familiar with the disaster plan or aware of pre-earthquake drills. Those without assigned roles often acted spontaneously, and those unaware of decision-making systems made more spontaneous decisions.

*At a disaster time, everyone needs to know their job. But I did not know my job: the scenario drove me to that job*.

##### Resourcefulness to Challenges to Individuals

As staff adapted to the new context, they divided teams in shifts, allowing everyone to take time off without interrupting services.

*We divided our team into two groups to cover days and nights. (…) One team would come in the daytime, do the cases, go home, and the other team would come in the nighttime*.

### Individual Resilience

Staff were essential to TUTH's adaptive capacity, but were constantly exposed to suffering and living a work-family dilemma. Interviewees recalled feeling fear, worry, and sorrow, sometimes even reporting “mental instability” and “depression”; but they also reported feeling pride and love for TUTH, and even recalled feeling “extra powers” to work. Inactivity or difficulties in contacting relatives often promoted negative thoughts, and “overworking” was a frequent strategy to avoid this.

*You try to compensate with overworking or doing something else, so that you do not feel depressed*.

Some interviewees were trying to forget their experiences or denying the possibility of future events, but did not elaborate on this during the interviews despite different probes. Such interviewees had apparently implemented fewer adaptations at home, and were less confident about their experiences.

This type of disaster is not happening again and again, I pray the god. Because that time was terrible, everyone was panicking, crying, things were destroyed …

#### Determinants of Individual Resilience

We identified three major determinants of hospital staff resilience: safety, meaningfulness, and sense of belonging.

##### Safety

Feeling safe helped managing negative feelings: for instance, knowing TUTH was earthquake-resistant allowed staff to continue working despite recurrent aftershocks. At home, interviewees reported improving safety by retrofitting and preparing emergency bags. The absence of safety and disaster prevention measures on a national level contributed to a persistence of fear until the time of the interview, and challenged recovery.

*We were terrified, but we knew that we were safe in the ICU, because that building was safe*.

*Now at this moment I can't say I am fully recovered. Because I feel scared of the roads, because roads are not properly constructed*.

##### Meaningfulness

Many interviewees felt their work was essential and contributed to a selfless aim, which helped them making sense of the tireless work and putting family second. Interviewees alienated from this meaning often felt trapped in their jobs and frustrated with the administration. Professional visibility seemed to influence this: technicians often felt overlooked and questioned the sense of their dedication, while those who directly cared for victims, or who had been recognized by the hospital, more often found their experiences meaningful.

One of my cousins, (…) he was found dead. (…) I couldn't even get there to say a few words on their sorrow and grief. What sort of job do I have, I am not even free to go there and express my sorrow!

*Now they have understood our importance. If there is work to do, they call us. If there is a meeting (…), they call us. Before, they never cared*.

##### Sense of Belonging

In order to focus on work without feeling guilty, hospital workers often left loved ones with extended families or friends, which was often described as a relief. Interviewees were proud of the family cohesiveness in Nepal, which they thought largely contributed to success. Extended community support also contributed to an inspiring sense of belonging. Another example was team spirit, as co-workers comforted each other, and organized shifts so all could spend time with their families.

*After the 2nd day I shifted my family to my uncle's house (…). They had like a family get-together. And I was free to work*.

*In my house, there were 3–4 families: doctors from this hospital, whose house had been damaged, they were in my house. We were like in a big hostel. (…) So time passed gossiping. (…) it was an enjoyable time actually*.

## Discussion

Our study was among the first to apply a hospital resilience framework in a post-disaster setting using staff experiences. We illustrated how the earthquake generated a complexity of events that challenged a tertiary hospital in Nepal, many of which were addressed with emerging adaptations. We found that functional challenges, not material, were major disruptors of hospital resilience, and because they are highly dependent on human behavior, individual staff resilience plays a major role.

Our analysis captured a richness of experiences that did not exactly fit in the proposed definitions of the “4R” dimensions. The theoretical and empirical literature regarding hospital system resilience has been mostly developed in pre-disaster contexts, and has barely been validated by field evidence from disaster settings (11). Although established plans did influence TUTH's resilience, our results emphasize the important role of emerging adaptations in the overall performance of the hospital, but the 4R framework does not differentiate emerging adaptations from pre-disaster preparedness, even if they are the only solutions as new, unforeseen problems arise. While more recent hospital disaster resilience frameworks recognize the importance of “adaptive flexibility” (25) or “adaptive capacity” (26), these concepts remain vaguely defined in the scientific literature, as they are mostly developed for the purposes of preparedness and do not include actual experiences from real disasters.

But, even if spontaneous adaptations are inevitable, it is important that they do not substantially reduce quality of care. Crises understandably lead to altered standards of care, but decision-making should be based on harmonized strategies (27), not on individual assumptions. We found that professionals with managerial roles or closer to the administration were more familiar with the disaster plan. The design of new disaster plans should be fed from the earthquake experiences of hospital staff, the frontline workers who will be executing the plan in the event of a disaster. Training all the staff on a regular basis should also be foreseen in such plans, to ensure everyone is familiar with the organizational procedures and knows the decision-making processes.

Foreign Medical Teams were sometimes perceived as an invasion undermining local capacity, but they were also useful to fill specific gaps. In Nepal, international teams were coordinated by the Health Emergencies Operations Center under the Ministry of Health, and they followed the classification and standards set by the World Health Organization (28). To ensure uniformity in treatment and referral, all EMTs were asked to use national protocols while managing cases. Furthermore, they were instructed to maintain detailed documentation for trauma and amputation patients who required follow-up and rehabilitation, and reported daily to the Ministry of Health. A report on the effectiveness of these teams in Nepal highlighted a weak coordination and lack of needs-based deployment. The coordination of Foreign Teams, in Nepal and elsewhere, should avoid overlap with local capacitated teams, and should ensure a real discussion of needs and strategies with the local health service administrations (29).

Finally, we demonstrated the importance of staff in ensuring continuity and quality of services. Previous studies have shown that hospital workers undergo a double burden during disaster response, and struggle between responsibilities toward their family and hospital duties (3, 30). We found that interviewees were generally more positive when they felt safe, attributed a meaning to their experiences, and felt a sense of belonging to a supportive community. A previous study with hospital staff after Typhoon Haiyan also had identified the importance of feeling safe and feeling committed toward the patients for staff to continue working; an interesting difference was the consistent reference to faith as a coping mechanism, which was absent in our sample (3). Other qualitative studies have shown the importance of making sense of a difficult experience to believe efforts were worthwhile (31); and the feeling of safety is a recurrent need expressed by health and humanitarian workers in times of emergencies (32–34). According to the Sense of Coherence, when an individual perceives a difficult situation as meaningful, he/she will feel it is worth the commitment, rather than a burden (31).

Our findings suggest that hospital resilience and individual staff resilience are interdependent, as the conditions at the hospital affect the individual, and the individual's performance influences the effectiveness of adaptations. Strategies that contribute to staff wellbeing in times of disaster response contribute to hospital resilience. International initiatives such as the “Hospitals Safe from Disasters” Campaign (35) or the HOPE network (18) should make this explicit, and this should also be translated in hospital disaster plans in Nepal and beyond, as the ongoing COVID-19 pandemic showed these issues are present across different types of health services and different types of countries. One possible way to improve adherence to and effectiveness of hospital disaster plans and training exercises is to include different professions, seniority, and functions of staff in their design, which also promotes disaster government at local levels and contributes to local community empowerment (36). Contributions of young people also can be promising and forward looking when regarding disaster resilience, and could be a valuable asset in the design of local hospital plans (37). Finally, co-design of strategies that help individuals make sense of difficult experiences, like organizing debriefing sessions, is a promising solution (32). Staff should also be given the space to work as it may be a coping strategy that helps to make the most of their competences and capacities, and to feel empowered.

### Limitations

The selection of interviewees, while diverse, may have missed some specific roles which could have enriched our analysis, such as emergency nurses. A selection bias may also be present, since interviewees were contacted by the hospital director and administrative chief. This may have also inhibited interviewees to fully express themselves. Because interviews were conducted three years after the earthquake, testimonies are subject to recall bias, but we believe its influence was minimal given the magnitude of the event. In addition, language and cultural biases may have influenced our results. We had planned to validate our results through a participative approach with hospital staff, but the COVID-19 pandemic prevented this. Finally, TUTH has a Japanese-donated earthquake-strengthened structure and is a tertiary, University hospital, and findings must be handled carefully before generalizing to other settings. However, we have applied common strategies that improve transferability to other settings, including a rigorous and transparent methodology, and allowing for unexpected issues to emerge rather than force fitting them in theoretical frameworks (38).

## Conclusions

Hospital resilience results from emerging and planned adaptations that prevent substantial loss of function and promote a quick re-start of interrupted activities. Frameworks and plans to improve hospital resilience must reflect flexibility of response. Individual resilience of hospital staff and hospital resilience are interdependent. In emergencies, concern for hospital staff welfare is central for sustainable disaster response and improved resilience.

## Data Availability Statement

The data used for this study is not readily available because it contains identifiable information. For more information please contact maria.rodrigues@uclouvain.be.

## Ethics Statement

This study involved human participants, in this case staff from the hospital. The study was reviewed and approved by the Ethics Committee of the Tribhuvan University's Institute of Medicine, case number 381(6-11-E)2/074/075. The participants provided their written informed consent to participate in this study.

## Author Contributions

MMA, JvL, DPM, DGS, and IA conceived the study. MMA, DGS, and IA obtained research funding. MMA, SST, KCK, and DPM supervised the conduction of the interviews and data collection. KCK and DPM recruited participants. MMA conducted and recorded the interviews, transcribed interviews conducted in English, and sent transcripts to all interviewees for validation. MMA coded the data and discussed themes with IA and JvL. MMA drafted the manuscript and all authors contributed to its revision. MMA took responsibility for the paper as a whole. All authors contributed to the article and approved the submitted version.

## Conflict of Interest

The authors declare that the research was conducted in the absence of any commercial or financial relationships that could be construed as a potential conflict of interest.
