# Supplementary material for: Hospital Resilience After the 2015 Earthquake in Nepal: Results From Semi-structured Interviews With Hospital Staff
Source: Front Public Health. 2021 Feb 26;9:602509. doi: 10.3389/fpubh.2021.602509 (PMC7952522; doi:10.3389/fpubh.2021.602509)
Supplement: Supplementary file 1 [file Table_1.DOCX]

**Supplementary file 1: *Interview guide***

Initials: _________________ Date: ____________________

Start time: ________________ End time: _______________________

*My name is Maria Moitinho de Almeida, I am a public health researcher working in Belgium. Based on previous research, my team understood that it is important to get an insight/better understanding of the impact of the 2015 Earthquake at a personal and work level.*

*I would like to ask you questions related to your personal experience in this hospital after the earthquake. We would like to record the interview if that is fine with you, which will only be used to help us remember all that you said.*

*Please read carefully the following consent form, and if you agree, you can sign and we can start with the interview.*

*Feel free to share any related idea that you may find important. Let’s start.*

Personal information

- - Sex : F / M / ………….
  - Age : ……………………
  - What was your work at TUTH during/after the earthquake ? …………………………….

*I will now ask you some questions about your experience after the earthquake on a personal, professional and organizational level.*

| Main questions | Additional questions | Clarifying questions |
| --- | --- | --- |
| - **Can you recall the time of the earthquake, and what it was like for you?**   *(Day of the earthquake and time immediately after)* | - Where were you on the day of the earthquake? - How did this event affect you emotionally / affect your life? - How did you experience your professional obligations? - How did you perceive your ability to work after the Earthquake? - What were the personal working conditions like? - How did your situation at home affect your ability to work? - How did you feel the hospital was functioning? What were the main challenges and difficulties you witnessed? - How do you think the quality of care was influenced in your department/hospital? | - Can you expand a little on this? - Can you tell me anything else? - Can you give me some examples? |
| - **If you think about the time between the earthquake and now, can you explain what were your main experiences?**   *(Transition)* | - How has your personal situation evolved since then? - How has your performance at the hospital evolved? - Can you recall main challenges and strategies to cope with a return to a routine? - How has hospital performed/functioned since then? |  |
| - **How do you feel the situation is now?**   *(Now)* | - How has this earthquake experience influence your personal and professional life? - How has your experience changed your current work behavior? - How do you feel the hospital is functioning now? - What are your impressions on the current situation now, compared to before? |  |
| *During this interview, some of the strong points were …. (Short summary)*   - **What are your impressions on the current situation, compared to before the Earthquake?**   *(Before)* | - Were you prepared to face such a disaster before the earthquake occurred? How do you feel now? (Personal and professional) - What were the main differences in the functioning of the hospital compared to now? |  |
| **Conclusion of Interview** | |  |
| - Are there any other issues regarding your experience in the hospital after the earthquake that we have not discussed but you would like to share?   OR   - Do you want to add anything regarding your experience during the response and recovery phases? | |  |
